# Evaluation of Patient Comfort and Impact of Different Anesthesia Techniques on the Temporomandibular Joint Arthrocentesis Applications by Comparing Gow-Gates Mandibular Block Anesthesia with Auriculotemporal Nerve Block

**DOI:** 10.1155/2022/4206275

**Published:** 2022-08-31

**Authors:** Onur Atalı, Elif Özçelik, Onur Gönül, Hasan Garip

**Affiliations:** ^1^Department of Oral and Maxillofacial Surgery, Faculty of Dentistry, Marmara University, Istanbul 34854, Turkey; ^2^Private Practice, Bursa 16000, Turkey

## Abstract

**Aim:**

Temporomandibular disorders (TMDs) are clinical situations that are characterized by pain, sound, and irregular movements of the temporomandibular joints. The most common method in the treatment of TMDs is arthrocentesis. This study aims to compare the effect of conventional extraoral auriculotemporal nerve block (ANB) and Gow-Gates (GG) mandibular anesthesia techniques on patient comfort in an arthrocentesis procedure.

**Materials and Methods:**

We performed this study on 40 patients who underwent TMJ arthrocentesis with ANB (*n* = 20) or GG (*n* = 20) mandibular anesthesia techniques at the Marmara University Faculty of Dentistry between 2016 and 2019. The predictor variable was the type of an anesthesia technique, and the outcome variables included were pain, maximum mouth opening (MMO), and protrusive movement (PM). They were compared at the preoperative period and 3^rd^ and 6^th^ month periods. Statistical analysis included means with standard deviations, a one-way ANOVA for continuous data, and the results were evaluated at the significance level of *p* < 0.05.

**Results:**

No statistically significant difference was observed between the VAS values, MMO, and PM averages of preoperative, 3^rd^ and 6^th^ months of ANB and GG (*p*=0.142, *p*=0.209, and *p*=0.148).

**Conclusion:**

Both anesthesia techniques have provided effective results in terms of pain and functional jaw movements in the postoperative period in arthrocentesis treatment.

## 1. Introduction

Temporomandibular disorders (TMDs) are a clinical situation characterized by pain, sound, and irregular movements of the temporomandibular joint. TMDs encompass functional changes and pathological conditions affecting the jaw and masticatory muscles. The most common method in the treatment of these disorders is arthrocentesis. Arthrocentesis of the temporomandibular joint (TMJ) was first described by DW Nitzan in 1991 [1]. It was based on washing out inflammatory mediators with lactated ringer solution with the help of two 18-gauge injector tips placed in the upper joint cavity. The main purpose of arthrocentesis is to remove the inflamed synovial fluid in the joint cavity, to provide the appropriate fluid viscosity, and to remove adhesions with the help of hydraulic pressure. The process is thought to reduce friction between articular surfaces, remove adhesions (lysis), and chemical mediators of pain and inflammation by lavage. It has been reported that the arthrocentesis method is the least invasive method with proven results. It has minimal potential risk of complications in patients with acute or chronic closed locking and is possibly an intermediate treatment method in the treatment of internal disorders. Arthrocentesis is widely used in the treatment of TMDs with a high success rate [2, 3].

TMJ is innervated by the auriculotemporal nerve. Arthrocentesis is usually performed under local anesthesia using an extraoral auriculotemporal nerve block (ANB). It is a simple and safe technique that blocks the auriculotemporal nerve along with the nervus alveolaris inferior (NAI) and nervus buccalis. The Gow-Gates block (GG) technique is a good alternative to the IANB technique and is generally used when it cannot provide adequate anesthesia with IANB [3]. However, onset of anesthesia may be slower in the Gow-Gates technique [4], and the frequency of anesthesia failure may be as high as in ANB until the clinician gains clinical experience [3].

This study aims to compare the effect of the conventional extraoral auriculotemporal nerve block (ANB) and GG mandibular anesthesia techniques on patient comfort in an arthrocentesis procedure in terms of pain, maximum mouth opening (MMO), and protrusive movement (PM).

## 2. Materials and Methods

### 2.1. Study Plan

This study was approved by the Ethics Committee (protocol number is 2020–412) of the Faculty of Dentistry of Marmara University and was conducted in accordance with the Helsinki Declaration. All subjects included in the study were informed about the study, and written informed consent was obtained from the subjects.

It was planned as a randomized, controlled, single blinded clinical trial. The study enrolled 40 patients at the Department of Oral and Maxillofacial Surgery of the Dentistry Faculty, Marmara University, Istanbul, Turkey, between 2016 and 2019. The study was conducted with 40 subjects. Extraoral auriculotemporal anesthesia was applied to 20 patients, and the mean age was 39.15 ± 10.35 in the control group (*n* = 20). Gow-Gates anesthesia was applied to 20 patients. The mean age was 37.6 ± 10.84 in the study group (*n* = 20) (Table 1).

After the patients read the information and consent form and agreed to participate in the study, a tracking number was given to each patient. Then all the patients were distributed to the groups by giving a randomized number generator by a second researcher.

The criteria for inclusion were as follows: the patients were between the ages of 18 and 65, systemically healthy, have had anterior disc displacement without reduction (ADDWoR) which was diagnosed with magnetic resonance imaging (MRI), and had no pathological formation in the TMJ region, and no arthrocentesis was previously performed.

The criteria for exclusion from the study were as follows: patients' refusal to be included in the study, having any disease causing inadequate wound healing, prolonged bleeding time, having any disease or drug usage affecting platelet structure function, and a history of allergy to the local anesthetic agent used.

### 2.2. Anesthesia Procedures

Intra-articular and pericapsular local anesthesia (40 mg/ml articaine hydrochloride + 0,012 mg/ml adrenaline hydrochloride; 2 ml; Fullcain® Fort Onfarma, Samsun, Turkey) was applied for the ANB of patients in Group 1, while Gow-gates mandibular anesthesia was performed for patients in Group 2.

Before the treatment, MRI was taken from TMJ regions of the patients in both open and closed positions from both sides. After these images were evaluated by a radiologist, the type of disc displacement was determined. Patients with ADDWoR were included in the study, and their treatment was started after completing their consent forms.

The auriculotemporal nerve is the terminal branch of the trigeminal nerve. The mandibular nerve, which is the third division of the trigeminal nerve, passes through the foramen ovale and exits from the base of the skull and then continues in the infratemporal fossa where it divides into two branches around the middle meningeal artery [5]. These branches are known as anterior and posterior trunks.The posterior trunk branches into the auriculotemporal nerve [6]. This nerve supplies cutaneous sensitivity to the auriculotemporal area including the external acoustic meatus, tragus, anterior portion of the ear, temporal scalp, posterior portion of the temple, tympanic membrane, TMJ capsule, and parotid gland. ANB is applied by injecting 5 mL of local anesthesia 1.5 cm in front of the ear at the level of the tragus [7, 8].

While performing Gow-Gates anesthesia, two extraoral points were determined in patients, the apex of the intertragic notch and the lower border of the tragus. The patients were asked to widely open her/his mouth. The extraoral landmark is the imaginary line drawn from the intertragic notch to the corner of the mouth. Injection is administered parallel to this line. The needle is intraorally located just below the mesiopalatal cusp of the maxillary 2^nd^ molar tooth [9, 10].

In this technique, the target region is the lateral aspect of the condylar neck, which is close to the pterygoid fovea. Thus, the solution is stored at a superior level than the conventional IAN block. The solution is then diffused in the inferior direction and comes towards the anterior direction, up to the pterygomandibular space and the buccinator muscle. Thus, all sensory branches of the mandibular nerve up to the mylohyoid nerve are exposed to anesthetic solution [11].

In our study, after applying different anesthesia techniques to patients with internal derangements in the TMJ region in two groups, arthrocentesis was performed, and then, occlusal splints were applied immediately after the operation which were named as combination therapy; the parameters of pain, maximum mouth opening (MMO), and protrusive movement (PM) were compared at the preoperative period and 3^rd^ and 6^th^ month periods.

### 2.3. Treatment Procedure and Recordings

Stabilization splints were prepared for each patient prior to the arthrocentesis operation and were applied immediately after the procedure. Stabilization splints were produced from hard acrylic resin for canine protection in lateral and protrusive jaw movements and with maximum contact in centric occlusion. The patients were recommended that they should use their splints in the range of 8–10 hours a day for 6 months after arthrocentesis and overnight. Intraoral controls of the splints were checked periodically.

**Figure 1:**
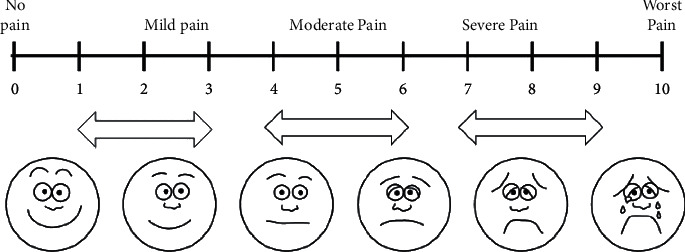
Visual Analogue Scale (0 = no pain, 1–3 = mild pain, 4–6 = moderate pain, 7–9 = severe pain, and 10 = unbearable).

Maximum mouth opening is the distance from the incisal edge of the upper central incisor to the incisal edge of the lower central incisor in the opposing arch, as measured with a flexible ruler when the patient is forced to open their mouth the most. The visual pain scale, on the other hand, is a visual recording technique that has figures and numbers on it and allows the patient to define their own pain level (Figure 1). It is filled by the patient at different times before and after the procedure. These two parameters are the main markers in determining the comfort of the arthrocentesis procedure in patients diagnosed with ADDWoR. These records are taken from all patients who have been examined and treated with the complaint of the TMJ irregularity in our clinic and are kept in the archive of our department.

### 2.4. Statistical Analysis

In this study, statistical analyses were performed with NCSS (Number Cruncher Statistical System) 2007 statistical software (Utah, USA) package program. In the evaluation of the data, besides the descriptive statistical methods (mean, standard deviation, median, and interquartile range), the Shapiro–Wilk normality test was used to analyze the distribution of variables, paired one-way variance analysis was used for time comparsions of variables with normal distribution, the Newman– Keuls multiple comparison test was used for subgroup comparisons, the independent *t*-test was used for comparison of binary groups, the Friedman test was used for time comparisons of variables that did not show normal distribution, Dunn's multiple comparison test was used for subgroup comparisons, the Mann–Whitney *U* test was used for comparison of binary groups, and the chi-square test for comparison of qualitative data. The results were evaluated at the significance level of *p* < 0.05.

## 3. Results and Discussion

No statistically significant difference was observed between the MMO averages of the preoperative (*p*=0.14) period and 3^rd^ (*p*=0.209) and 6^th^ (*p*=0.148) months of the ANB and the GG groups (Table 1).

A statistically significant change was observed between the preoperative, 3^rd^, and 6^th^ month MMO averages of Group 1 (*p*=0.0001), while preoperative MMO averages were found to be statistically significantly lower than the average of the 3^rd^ and 6^th^ month groups (*p*=0.0001); no statistically significant difference was observed between the 3^rd^ and 6^th^ month averages (*p*=0.102) (Table 2).

A statistically significant change was observed between the preoperative, 3^rd^, and 6^th^ month PM averages of Group 2 (*p*=0.0001). Preoperative PM averages were found to be statistically significantly lower than PM averages of the 3^rd^ and 6^th^ month values (*p*=0.0001); the mean of the 3^rd^ month PM values was found to be statistically significantly lower than the average of the 6^th^ month period (*p*=0.030) for Group 2 (Table 2).

No statistically significant difference was observed between the preoperative, 3^rd^, and 6^th^ month VAS values of Group 1 and Group 2 (*p*=0.474, *p*=0.487, and *p*=0.267). A statistically significant change was observed between the preoperative, 3^rd^, and 6^th^ month VAS values of Group 1 (*p*=0.0001). However, the preoperative VAS values were found to be statistically significantly higher than VAS values of the 3^rd^ and 6^th^ month periods (*p*=0.0001); no statistically significant difference was observed between the 3^rd^ and 6^th^ month periods (*p*=0.437) for Group 1 (Table 2).

When comparing the preoperative period with other time periods, there was no statistically significant difference between the ANB and the GG groups in terms of MMO, PM, and VAS pain parameters (Table 3).

## 4. Discussion

TMJ arthrocentesis is a simple and minimally invasive surgical method for the treatment of TMJ disorders. The main aim of arthrocentesis is to remove inflammatory mediators from the synovial fluid of the joint cavity, break adhesions, reduce pain, and increase joint mobility. It is used as a treatment option both for the displacement of the articular disc and for degenerative inflammatory joint disorders [2, 12].

The success rate of arthrocentesis varies between 70% and 90% [13–16]. Many studies have stated that treatments should start from conservative ones such as occlusal splints and muscle relaxants in the TMJ internal derangements, and surgical therapy is foreseen to be used in cases of failure of these treatments and especially when arthropathy is persistent. In most of the clinical studies, we recommend combination therapy that is combined of arthrocentesis and occlusal splint applications [17–21]. In some studies, arthrocentesis is used as the first treatment option in internal derangements of TMJ [13, 18, 21]. In the present study, the authors have applied the combination therapy.

In ADDWoR patients, conservative treatment using only occlusal splints can sometimes be successful. However, it is troublesome for some patients because it is necessary to use splints for very long periods. According to some studies, only occlusal splints have no advantage in ADDWoR treatment [12].

Since all studies related to arthrocentesis in the literature are performed by the ANB technique, we can only compare the data of these studies with the control group of the present study. When comparing the preoperative period with 3^rd^ and 6^th^ months, there was no statistically significant difference between the ANB and the GG groups in terms of MMO, PM, and VAS pain parameters in our study. Heo et al. [22] and Ghanem [23] found that MMO and PM values were statistically higher in the 6^th^ month compared to the preoperative period. The results of the present study reflect these findings.

Abbasgholizadeh et al. [24] showed that MMO after arthrocentesis increased significantly in the 1^st^ month in the combination therapy group, but these values disappeared starting from the 3^rd^ month, and the mean painless mouth opening amount was over 35 mm in the 6^th^ month. In the same study, a significant decrease was observed in the follow-up evaluations of VAS pain scores from the 1^st^ month in the combination therapy group. However, Ghanem [23] showed that VAS pain scores decreased significantly after 1^st^ month after arthrocentesis, and this decreased pattern continued in the controls at the 3^rd^ and 6^th^ months. In this study, the VAS pain scores have decreased with time, which confirms the findings in the literature.

Nishimura et al. [25] described the MMO value of more than 38 mm, and mild or no pain scores were counted as successful procedures in their study. Bas et al. [26] accepted the MMO value of more than 35 mm and VAS pain scores of lower than 3 were accepted as successful at the 3^rd^ month follow-up in their study. When considered the success rate of pain and MMO values independently, it was assessed as 91% and 79.5%, respectively.

Many researchers have applied arthrocentesis under local or general anesthesia. Ziegler et al. [27] used bupivacaine in their patients to reduce pain during and after the operation. General anesthesia has been reported to be more comfortable since arthrocentesis is a painful procedure [28]. In another study, it was suggested to apply arthrocentesis with local anesthesia due to complications of general anesthesia [29]. Emes et al. [30] reported in their case series consisting of 24 patients that there is no need to use Gow-Gates anesthesia for the auriculotemporal nerve block in arthrocentesis.

Clinical arthrocentesis studies showed that the VAS pain score values were statistically significantly higher in the preoperative period than those in the 6^th^ month [3, 20–24, 31, 32]. The mean preoperative VAS score of this study was 7.10 ± 1.37 for ANB and 7.30 ± 1.38 for GG, which were found to be statistically significantly higher than those of the 6^th^ month 2.30 ± 2.2 and 1.60 ± 1.31, respectively (*p*=0.0001). Our results are similar to the findings of the previous studies.

Madan et al. [33] have compared the clinical efficacy, degree of patient acceptability, advantages, disadvantages, and limitations of the classical and the Gow-Gates techniques for providing anesthesia in patients undergoing bilateral symmetrical surgical removal of impacted mandibular third molar under local anesthesia. They concluded that GG is found to be more reliable, beneficial, and has a higher success rate than a classical inferior alveolar nerve block technique while ignoring delayed onset of anesthesia of the Gow-Gates technique.

## 5. Conclusion

In conclusion, both anesthesia techniques have provided effective results in terms of pain and functional jaw movements in the postoperative period in arthrocentesis treatment. In our study, applying the Gow-Gates mandibular anesthesia instead of the conventional auriculotemporal anesthesia techniques used in TMJ arthrocentesis may be more advantageous in terms of patient comfort. Although the results are close to each other and no significant differences are seen in both groups, we recommend applying the Gow-Gates anesthesia technique in routine arthrocentesis due to the patient's comfort during the anesthesia procedure.

## Figures and Tables

**Table 1:** Mean age, gender, maximum mouth opening (MMO), protrusive movement (PM), and VAS pain distribution of auriculotemporal nerve block (ANB) and Gow-Gates (GG).

			ANB		GG		*p*
Age		Mean ± SD	39.15 ± 10.35		37.6 ± 10.84		0.646^*∗*^
Gender		Male	13	65.00%	9	45.00%	0.204+
		Female	7	35.00%	11	55.00%	
Max. mouth opening	Preop	Mean ± SD	33.85 ± 5.72		36.55 ± 5.67		0.142^*∗*^
	3^rd^ Mt	Mean ± SD	36.75 ± 6.39		39.15 ± 5.44		0.209^*∗*^
	6^th^ Mt	Mean ± SD	37.30 ± 5.73		39.85 ± 5.17		0.148^*∗*^
	pǂ		0.0001		0.0001		
Protrusive mov.	Preop	Mean ± SD	5.40 ± 1		5.65 ± 1,18		0.474^*∗*^
	3^rd^ Mt	Mean ± SD	6.20 ± 1,36		6.50 ± 1,32		0.483^*∗*^
	6^th^ Mt	Mean ± SD	6.40 ± 1,14		6.80 ± 1,11		0.267^*∗*^
	pǂ		0.0001		0.0001		
VAS	Preop	Mean ± SD	7.10 ± 1.37		7.30 ± 1.38		0.689^†^
		Median (IQR)	7 (6–8)		7 (6–8)		
	3^rd^ Mt	Mean ± SD	2.60 ± 1.76		1.80 ± 1.51		0.107^†^
		Median (IQR)	2 (1–3.75)		1 (1–2.75)		
	6^th^ Mt	Mean ± SD	2.30 ± 2.2		1.60 ± 1.31		0.414^†^
		Median (IQR)	2 (0–3.75)		1.5 (1-2)		
	pǂ		0.0001		0.0001		

^*∗*^Independent t-test, ^†^Mann–Whitney U-test, ^ǂ^one-way variance analysis, ^‡^Friedman test.

**Table 2:** ^ǂ^Newman–Keuls multiple comparison test after one-way variance analysis and ^‡^Dunn's multiple comparison test after the Friedman test.

	Max. mouth opening ^ǂ^		Protrusive movement ^ǂ^		VAS^‡^	
	ANB	GG	ANB	GG	ANB	GG
Preop/3^rd^ Mt	0.0001	0.0001	0.0001	0.0001	0.0001	0.0001
Preop/6^th^ Mt	0.0001	0.0001	0.0001	0.0001	0.0001	0.0001
3rd/6^th^ Mt	0.102	0.015	0.330	0.030	0.437	0.214

**Table 3:** Difference values of ANB and GG between preoperative and 3^rd^ and 6^th^ months.

Difference values		ANB	GG	*p* ^†^
MMO 3^rd^ Mt-preop	Mean ± SD	2.90 ± 1.97	2.60 ± 1.60	0.573
	Median (IQR)	3 (1–5)	2 (1,25–4)	
MMO 6^th^ Mt-preop	Mean ± SD	3.45 ± 1.85	3.30 ± 1.38	0.804
	Median (IQR)	3 (2.25–5)	4 (2–4)	
PM 3^rd^ Mt-preop	Mean ± SD	0.8 ± 0.83	0.85 ± 0.67	0.645
	Median (IQR)	1 (0-1)	1 (0-1)	
PM 6^th^ Mt-preop	Mean ± SD	1.00 ± 0.8	1.15 ± 0.81	0.546
	Median (IQR)	1 (0–2)	1 (0,25–2)	
VAS preop-3^rd^ Mt	Mean ± SD	4.50 ± 1.82	5.5 ± 1.91	0.088
	Median (IQR)	5 (2,25–6)	5.5 (4–7)	
VAS preop-6^th^ Mt	Mean ± SD	4.80 ± 1.96	5.70 ± 1.66	0.145
	Median (IQR)	5 (3–6.75)	6 (4.25–7)	

^†^Mann–Whitney U-test.

## Data Availability

The datasets used and analyzed during the current study are available from the corresponding author on reasonable request.
